# Predicting Falls in People with Multiple Sclerosis: Fall History Is as Accurate as More Complex Measures

**DOI:** 10.1155/2013/496325

**Published:** 2013-09-26

**Authors:** Michelle H. Cameron, Emily Thielman, Rajarshi Mazumder, Dennis Bourdette

**Affiliations:** ^1^Department of Neurology, Oregon Health & Science University and Portland VA Medical Center, Portland, OR 97219, USA; ^2^National Center for Rehabilitative Auditory Research, Portland VA Medical Center, Portland, OR 97219, USA; ^3^Oregon Health & Science University, Portland, OR 97219, USA

## Abstract

*Background.* Many people with MS fall, but the best method for identifying those at increased fall risk is not known. *Objective.* To compare how accurately fall history, questionnaires, and physical tests predict future falls and injurious falls in people with MS. *Methods.* 52 people with MS were asked if they had fallen in the past 2 months and the past year. Subjects were also assessed with the Activities-specific Balance Confidence, Falls Efficacy Scale-International, and Multiple Sclerosis Walking Scale-12 questionnaires, the Expanded Disability Status Scale, Timed 25-Foot Walk, and computerized dynamic posturography and recorded their falls daily for the following 6 months with calendars. The ability of baseline assessments to predict future falls was compared using receiver operator curves and logistic regression. *Results.* All tests individually provided similar fall prediction (area under the curve (AUC) 0.60–0.75). A fall in the past year was the best predictor of falls (AUC 0.75, sensitivity 0.89, specificity 0.56) or injurious falls (AUC 0.69, sensitivity 0.96, specificity 0.41) in the following 6 months. *Conclusion.* Simply asking people with MS if they have fallen in the past year predicts future falls and injurious falls as well as more complex, expensive, or time-consuming approaches.

## 1. Introduction

Multiple sclerosis (MS) impairs cognition, muscle strength, muscle tone, sensation, coordination, and gait, all of which are associated with an increased risk for falls [1, 2]. A number of studies demonstrate that people with MS fall frequently [3–7] and suffer from various sequelae of falls, including injury [5, 8, 9] and death [10], fear of falling, and reduced participation in occupational and leisure activities [11, 12]. To appropriately initiate fall prevention interventions, one must be able to identify those people with MS at increased risk for falls. Although various physical tests and questionnaires correlate with fall risk in MS [13–16], the ideal measure, which is quick, easy, and has high predictive accuracy, has not been identified.

The simplest, quickest approach for predicting future falls is to ask about past falls, but the strength of the relationship between past and future falls, and as well as predictive performance in comparison to other more complex measures in MS, is not known. In this study we compared how well future falls could be predicted by past falls, questionnaires, clinical measures readily performed by a physician, and computerized dynamic posturography (CDP), the gold-standard measure of human standing balance and postural control. 

The objective of this study was to identify, in subjects with MS, which of the following had the highest average sensitivity for predicting a fall or an injurious fall in the subsequent 6 months: a history of falls within the past year; responses to the Activities-specific Balance Confidence (ABC), Falls Efficacy Scale-International (FES-I) and Multiple Sclerosis Walking Scale-12 (MSWS-12) questionnaires; Expanded Disability Status Scale (EDSS) and Timed 25 Foot Walk (T25-FW) scores; Automatic Postural Response (APR) latency from CDP testing. 

## 2. Materials and Methods

### 2.1. Standard Protocol Approvals, Registrations, and Patient Consents

**Figure 1:**
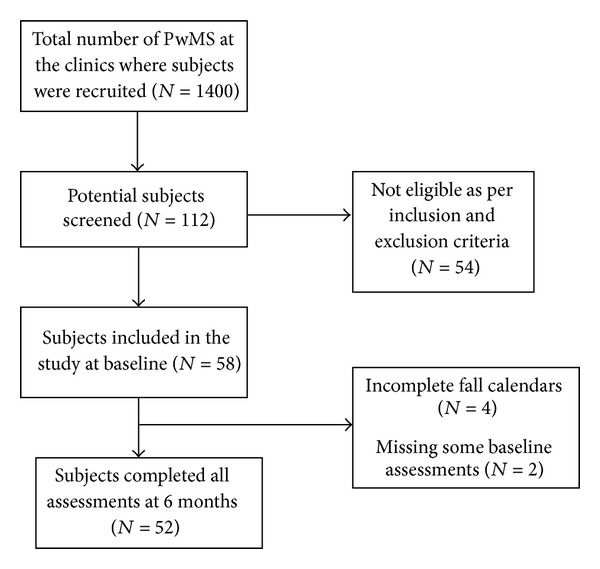
Participant flow diagram.

 This was a prospective cohort study carried out at a Department of Veterans' Affairs and an academic medical center in the Northwest USA. The Institutional Review Board at both centers approved the protocol, and written informed consent was obtained from all patients participating in the study. The subjects were 52 people with MS recruited from the outpatient MS specialty clinics of these centers and surrounding community neurology clinics in 2010 to 2011. Potential subjects were recruited using flyers posted at the clinics, by providing information at patient education programs and support groups, and by referral from clinic healthcare providers. The clinics serve a total of approximately 1400 people with MS annually. From this population, 112 potential subjects expressed interest in participating in the study and, of these, 58 met inclusion criteria and consented to participate. Fifty two subjects completed all measures analyzed and were included in this analysis (Figure 1).

### 2.2. Inclusion and Exclusion Criteria

 Inclusion criteria were age from 18 to 50 years, clinically and MRI-confirmed diagnosis of MS (McDonald criteria 2005 [17]) of any subtype, mild-to-moderate MS-associated disability (EDSS score ≤ 6.0 [18]), willing and intellectually able to understand and sign an informed consent and adhere to protocol requirements, able to complete a written daily record of falls for 6 months, community dwelling, and no clinically significant MS relapse within 30 days prior to baseline testing. Exclusion criteria were a self-reported condition other than MS known to affect balance or gait, unable to follow directions in English, unhealed fractures or other conditions conveying risk of injury during balance testing; blindness, or, unable to walk more than 100 meters. 

### 2.3. Variables

#### 2.3.1. Falls

Falls and injurious falls in the 6 months following the baseline assessment were assessed prospectively by subjects documenting their falls each day for 6 months on monthly fall calendars and returning these calendars at the end of each month. The calendar stated, “Please write in the number of falls you have each day. A *fall* is any unexpected event that results in you ending up on the ground, floor, or any lower surface.” In addition, subjects were asked to document each month if they suffered any injuries as a result of any fall. Subjects were contacted by phone during the first week of the fall count to reminder them to count their falls and to answer any calendar-related questions. Subjects returned the completed fall calendar at the end of each month and if the calendar was not received within 1 week after the end of the month, subjects were contacted by phone to ask for the calendar to be sent in.

The history of falls over the past 2 and 12 months was assessed at baseline with a questionnaire designed specifically for this study that included the following questions: How many times have you fallen in the past 2 months? How many times have you fallen in the past year? Options for responses were 0, 1, 2, 3, 4, 5–10, and more than 10 times. 

#### 2.3.2. Subjectively Assessed Balance Confidence, Fear of Falling, and Walking

Balance confidence and fear of falling were assessed at baseline with the ABC and the FES-I questionnaires, respectively [19, 20]. Scores on these scales are associated with imbalance and fall risk in people with MS [13, 16, 21]. The ABC questionnaire asks subjects to rate their confidence in terms of whether they expect to lose balance or become unsteady when performing 16 mobility-related tasks. The FES-I questionnaire asks subjects to rate how concerned they are about falling when performing 16 activities of daily living. The MSWS-12 questionnaire was used to assess the self-reported impact of MS on walking [22]. The MSWS-12 questionnaire asks subjects to rate how much MS has limited 12 different aspects of their walking in the previous 2 weeks.

#### 2.3.3. Clinically Assessed Walking

The EDSS [18] and T25-FW [23] assessed overall MS-associated impairment and walking. The EDSS is an MS-specific measure scored based on a clinical neurological examination and the subject's walking capacity. EDSS scores are correlated with fall risk in MS [4, 21, 24]. The T25-FW is a component of the MS-specific clinical measure of disease-associated impairment, the Multiple Sclerosis Functional Composite (MSFC) [23]. For the T25-FW the subject is timed walking 25 feet as quickly as possible but safely. The relationship between T25-FW performance and fall risk has not been reported. 

#### 2.3.4. Computerized Balance Assessment

CDP objectively and precisely assessed standing balance performance. CDP uses commercially available specialized equipment to examine balance control. The subject stands on a force platform with a visual surround. During testing, the platform and/or the visual surround move under computer control and the platform measures the timing and force of the subject's postural responses. The delay between the platform moving backwards and the subject responding by exerting plantar flexion force on the platform is known as the automatic postural response (APR) latency. The APR latency is the most precisely measured and predominant balance abnormality in people with MS [25–29]. The relationship between APR latency and future fall risk in MS has not been reported. The composite APR latency from small, medium, and large backward translations was used for analysis in this study. 

### 2.4. Statistical Methods

The goal of the analysis was to identify which of the baseline questionnaire, clinical, and computerized measures had the highest sensitivity for predicting the occurrence of any falls or any injurious falls in the following 6 months. 

The probability of any falls or any injurious falls occurring in the 6 months following baseline assessment was modeled using logistic regression, with each predictor, including demographic characteristics and test performance, considered separately. Falls data from the calendars were categorized as zero and one or more falls or injurious falls during the entire 6-month follow-up period. Questionnaires were scored according to their scoring algorithms. The area under the receiver operating characteristic (ROC) curve (AUC) was computed from each logistic regression result. The ROC curve plots the true positive rate (predicted falls that actually occurred) against the false positive rate (predicted falls that did not actually occur). The AUC is the area under the ROC curve and provides a summary measure of the accuracy of the potential predictive variables. Thus, in this study, the AUC provides a measure of the average sensitivity for a specific baseline variable to predict a fall or an injurious fall within the following 6 months. The AUC ranges in value from 0.5 (chance) to 1.0 (perfect accuracy). Significance of the AUC measures was determined by repeated permutation testing. 

Multivariate prediction models that combined two or more test components (e.g., APR from posturography and FES questionnaire score) were also considered. Logistic regression was performed using all analysis variables, and a stepwise selection process was employed to find the model that best predicts falls and injurious falls within the following 6 months. The selection process narrowed down the analysis variables to those that best contributed to the prediction model. Sensitivity, specificity, and positive and negative predictive values were computed for the variable with the highest AUC.

## 3. Results

### 3.1. Participants

Two-thirds of the participants were female. At baseline, their average age was about 40 years (mean 39.8, range: 22 to 50) and the majority (94%) had relapsing-remitting MS (RRMS). The mean time since MS diagnosis was 6.3 years, the mean time since disease onset was 10.4 years, and the mean EDSS score was 2.8 (Table 1).

### 3.2. Falls and Test Performance

Thirty-seven (71%) subjects fell at least once in the 6 months following baseline assessment, with a range from 1 to 21 falls in this period. Twenty-three subjects (44%) sustained at least one injury from a fall during this 6-month period. The subjects who were injured by a fall sustained bruises, cuts and grazes, sprains, and strains, and one subject had a collapsed lung, ruptured spleen, and blood loss as the result of a fall. Many subjects reported having pain associated with their injuries and four subjects reported only pain as the injury associated with a fall (Table 2).

**Figure 2:**
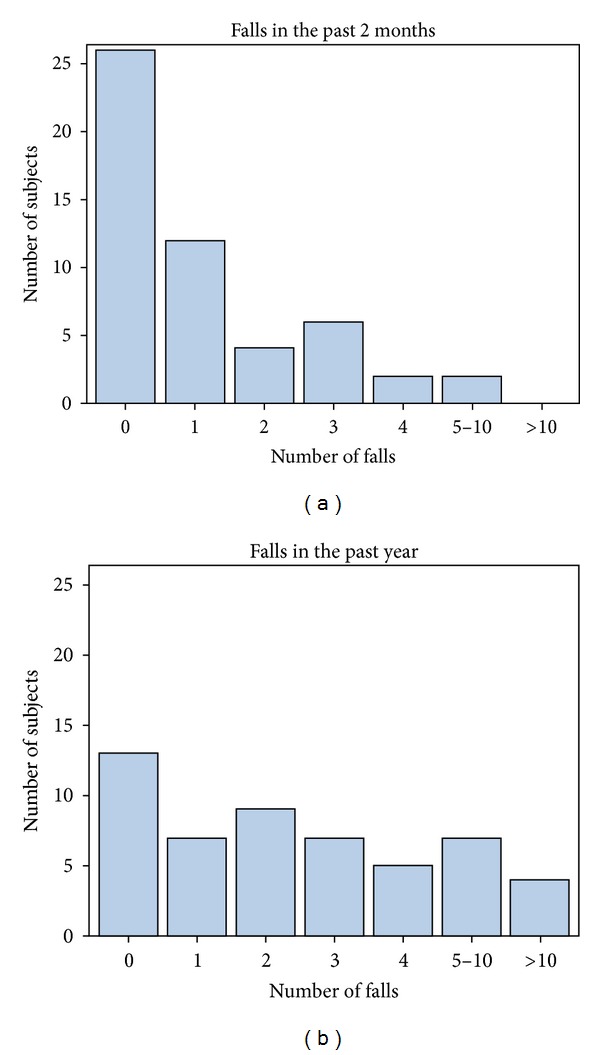
Number of falls sustained over the 2 months and 1 year prior to baseline.

Half of the subjects reported falling at least once in the 2 months prior to baseline. Of those who reported having fallen in the past 2 months, about half fell once and the rest fell 2 or more times. Three-quarters of the subjects reported falling at least once in the year prior to baseline, 7 subjects reported falling once in the past year, and 32 subjects reported multiple falls, including 4 who fell more than 10 times in the past year (Figure 2). 

The mean ABC score for the sample was 78.8 (SD 20.9), the mean FES-1 score was 25.8 (SD 9.6), the mean MSWS-12 score was 26.1 (SD 14.8), the mean 25-foot walking speed was 5.7 seconds (SD 3.8), and the mean APR latency was 135.7 milliseconds (SD 19.7) (Table 3). 

### 3.3. Main Results

#### 3.3.1. Prediction of Falls and Injurious Falls in the following 6 Months

The AUC computed from logistic regression, with each predictor, including demographic characteristics and test performance, considered separately or modeled together, demonstrated that falls in the past year had the highest sensitivity for predicting the occurrence of any falls or any injurious falls in the 6 months following baseline testing (AUC = 0.75 for falls, 0.69 for injurious falls). Among the individual potential predictors, the EDSS score had the lowest AUC (0.60) for predicting any falls and the APR latency had the lowest AUC (0.52) for predicting any injurious falls (Table 3).

The AUC is a measure of the average predictive sensitivity, or accuracy, of a predictive variable. The AUC measures the average true positive rate (predicted falls that actually occurred) over all false positive rates (predicted falls that did not actually occur). In this study, the AUC provides a measure of the average sensitivity for a specific baseline variable to predict a fall or an injurious fall within the following 6 months.

#### 3.3.2. Sensitivity, Specificity, and Predictive Value for Falls in the Past Year as Predictors of Falls and Injurious Falls in the following 6 Months

Reporting having one or more falls in the past year predicts that a subject will sustain one or more falls in the following 6 months with a sensitivity of 0.89 (95% CI 0.73–0.96) and a specificity of 0.56 (95% CI 0.31–0.79). The positive predictive value for this classification is 0.82 (95% CI 0.66–0.92), and the negative predictive value is 0.69 (95% CI 0.39–0.90). Reporting having one or more falls in the past year predicts that a subject will sustain one or more injurious falls in the following 6 months with a sensitivity of 0.96 (95% CI 0.76–1.00) and a specificity of 0.41 (95% CI 0.24–0.61). The positive predictive value for this classification is 0.56 (95% CI 0.40–0.72), and the negative predictive value is 0.92 (95% CI 0.62–1.00) (Table 4).

## 4. Discussion

This is the first prospective cohort study to demonstrate that, in people with MS, a history of a fall in the past year is a better predictor of falls and injurious falls in the following 6 months than questionnaires about balance or walking, clinical measures of MS-associated impairment or walking speed, or computerized dynamic posturography. Specifically, based on the data presented in this study, if a patient with MS reports falling in the past year they have an 82% probability of falling again in the following 6 months and a 56% probability of sustaining an injurious fall in the following 6 months. Equally importantly, if a patient with MS reports not falling in the past year, they have a 69% probability of not falling in the following 6 months and a 92% probability of not sustaining an injurious fall in the following 6 months. These test performance characteristics are well suited to clinical practice. The question, “Have you fallen in the past year?” is quick and easy to ask and accurately identifies those at high risk for falls and those not at high risk for injurious falls in the following 6 months.

The American Academy of Neurology Quality Standards Subcommittee practice parameter gives a level A recommendation for asking all patients in a neurology practice with fall risk factors about falls in the past year [1]. The fall risk factors identified in this practice parameter include disorders of gait and balance, use of assistive devices to ambulate, lower extremity weakness, or sensory loss. MS is not identified specifically as a fall risk factor but the parameter recommends that future research assesses the fall “risk for persons with specific neurologic conditions which may affect gait, mobility, or balance,” and “identify screening tools that may be performed quickly and easily in the office or at the bedside.” Prior falls have been found to be a strong predictor of future falls in older adults [30, 31] and in people with Parkinson's disease [32]. Our study demonstrates that prior falls are a strong predictor of future falls in MS. Our study also confirms the high frequency of falls in people with MS [7, 16, 24]. In our study 71% of subjects fell at least once in 6 months. Other studies prospectively assessing fall frequency in MS have reported 48% to 70% of subjects falling at least once in 3 to 12 months [7, 21, 24].

Limitations of this study include its moderate sample size, that it was carried out in a single location, that subjects were all aged 50 or under, that most (94%) of the subjects had relapsing remitting MS, and that falls were self-reported on a calendar. However, the sample size was sufficient to demonstrate statistically and clinically significant relationships, and previous studies indicate that the incidence, timing and location of falls in people with MS are similar in different parts of the world [33]. Generalizability of the findings is limited because subjects were recruited from tertiary care MS centers in a single geographic region and they were recruited for a study of imbalance in MS, potentially selecting for people with greater imbalance and fall risk than in the general population of people with MS. Constraining the age of the cohort helped isolate the effects of MS on balance and fall risk at the cost of limiting generalizability to older people with MS. The high proportion of subjects with relapsing remitting MS was unexpected but may be related to restricting the age of the cohort to under 50 and the EDSS to 6.0 or less, biasing away from primary and secondary progressive MS both of which are associated with a later age of onset and higher level of disability than relapsing remitting MS. Although self-reporting of falls by subjects could have biased our findings, because there is no accurate alternative to self-report, fall calendars are considered the gold standard for measuring falls prospectively [34]. Accelerometer-based fall detection methods are available, but these are limited by moderate specificity and poor sensitivity [35]. To assure data completeness, subjects in this study were contacted by phone to remind them to complete and return their fall calendars. Future studies would benefit from a larger sample size and a more diverse sample to enhance generalizability and to allow for further subgroup analyses.

One of the most important limitations of this study's findings is that predicting future falls based on previous falls requires the person to fall at least once. Ideally, one would predict and prevent a person's first fall. However, reassuringly, the data indicate that if a patient with MS reports not falling in the past year, although they may fall in the following 6 months, they are extremely unlikely to be injured by a fall in this time frame. In addition, only asking if a person has fallen in the past year will not identify the specific factors contributing to fall risk for the individual, which might be needed to select effective interventions to help prevent future falls. However, this simple approach quickly identifies those at increased fall risk for whom further discussion and testing may help direct selection of fall prevention interventions such as use of an assistive device, physical therapy assessment and treatment, medication changes, or multidimensional fall prevention education. In addition, just as MRI and relapse rate are used to select patients with active MS for trials of MS disease modifying therapy, this study suggests that a history of falls is an ideal tool for selecting those patients with MS at high risk for falls for participation in trials of fall prevention therapies.

## 5. Conclusions

In summary, simply asking people with MS if they have fallen in the past year predicts future falls and future injurious falls as well as more complex, expensive, or time-consuming approaches. In the context of a busy clinical practice this is feasible and certainly quicker and easier than other options. We recommend that clinicians ask all their MS patients whether they have fallen in the past year to easily identify those at increased risk for falling and for sustaining fall related injury. 

## Figures and Tables

**Table 1:** Demographics and sample characteristics (*n* = 52).

Variable	Value
Age: mean (SD, range)	39.8 (8.4, 22–50)
Gender *N* (%)	
Female	35 (67)
Male	17 (33)
MS subtype	
RRMS	49
SPMS	3
PPMS	0
PRMS	0
EDSS: mean (SD, median, range)	2.8 (1.5, 3.0, 0–6)
Disease duration from diagnosis in years: mean (SD, range)	6.3 (5.6, 0–22)
Disease duration from onset in years*: mean (SD, range)	10.4 (8.3, 0–31)

*For one subject (included in analysis) date of disease onset was unknown.

**Table 2:** Types of injuries reported by the 23 subjects who were injured by a fall in the 6 months evaluated.

Type of injury	Number reported*
Bruise	32
Cut or graze	25
Sprain or strain	3
Pain only	4
Other	3 (1 subject had a collapsed lung, spleen rupture, and blood loss as the result of a fall. 1 subject lost a fingernail as the result of a fall. 1 subject reported swelling associated with bruises, cuts or grazes, sprain or strain, and pain, all associated with a single fall)

*The number of injuries was greater than the number of subjects injured by a fall as subjects could be injured by a fall more than once in the period and subjects could report more than one injury as the result of single fall.

**Table 3:** Average sensitivity of baseline variables for predicting falls and injurious falls in the following 6 months.

	Occurrence/score	AUC: any falls in the following 6 months	AUC: any injurious falls in the following 6 months
A fall in the past year *n* (%)	39 (75%)	**0.75** ***P*** < **0.001**	**0.69** ***P*** < **0.001**
A fall in the past 2 months *n* (%)	26 (50%)	0.71 *P* = 0.002	0.64 *P* = 0.012
ABC total score	78.8 ± 20.9	0.69 *P* = 0.018	0.66 *P* = 0.025
FES-I total score	25.8 ± 9.6	0.66 *P* = 0.029	0.59 *P* = 0.14 (NS)
MSWS-12 total score	26.1 ± 14.8	0.69 *P* = 0.013	0.65 *P* = 0.031
APR latency from posturography (in milliseconds)	135.7 ± 19.7	0.62 *P* = 0.082	0.52 *P* = 0.40 (NS)
EDSS score	2.8 ± 1.5	0.60 *P* = 0.14 (NS)	0.55 *P* = 0.30 (NS)
T25FW time in seconds	5.7 ± 3.8	0.71 *P* = 0.009	0.64 *P* = 0.043

AUC: area under the curve; ABC: Activities-specific Balance Confidence; FES-I: Falls Efficacy Scale International; MSWS-12: Multiple Sclerosis Walking Scale-12; APR: Automatic Postural Response; EDSS: Expanded Disability Status Scale; T25FW: Timed 25-Foot Walk.

**Table 4:** Association between falls in the past year and falls or injurious falls in the following 6 months.

	Any falls in the following 6 months		Any injurious falls in the following 6 months	
	Yes	No	Yes	No
Fell in the past year				
Yes	32	7	22	17
No	4	9	1	12

## Glossary

ABC: Activities-specific balance confidence
APR: Automatic postural response
AUC: Area under the curve
CDP: Computerized dynamic posturography
EDSS: Expanded disability status scale
FES-I: Falls efficacy scale international
MS: Multiple sclerosis
MSFC: Multiple sclerosis functional composite
MSWS-12: Multiple Sclerosis Walking Scale-12
ROC: Receiver Operating Curve
T25-FW: Timed 25-Foot Walk.
